# CloVR-ITS: Automated internal transcribed spacer amplicon sequence analysis pipeline for the characterization of fungal microbiota

**DOI:** 10.1186/2049-2618-1-6

**Published:** 2013-02-04

**Authors:** James Robert White, Cynthia Maddox, Owen White, Samuel V Angiuoli, W Florian Fricke

**Affiliations:** 1 Institute for Genome Sciences, University of Maryland School of Medicine, BioPark II - 801 West Baltimore Street, Baltimore, MD, 21201, USA

**Keywords:** Internal transcribed spacer (ITS), Fungal microbiota, Automated sequence analysis pipeline, Cloud computing

## Abstract

**Background:**

Besides the development of comprehensive tools for high-throughput 16S ribosomal RNA amplicon sequence analysis, there exists a growing need for protocols emphasizing alternative phylogenetic markers such as those representing eukaryotic organisms.

**Results:**

Here we introduce CloVR-ITS, an automated pipeline for comparative analysis of internal transcribed spacer (ITS) pyrosequences amplified from metagenomic DNA isolates and representing fungal species. This pipeline performs a variety of steps similar to those commonly used for 16S rRNA amplicon sequence analysis, including preprocessing for quality, chimera detection, clustering of sequences into operational taxonomic units (OTUs), taxonomic assignment (at class, order, family, genus, and species levels) and statistical analysis of sample groups of interest based on user-provided information. Using ITS amplicon pyrosequencing data from a previous human gastric fluid study, we demonstrate the utility of CloVR-ITS for fungal microbiota analysis and provide runtime and cost examples, including analysis of extremely large datasets on the cloud. We show that the largest fractions of reads from the stomach fluid samples were assigned to Dothideomycetes, Saccharomycetes, Agaricomycetes and Sordariomycetes but that all samples were dominated by sequences that could not be taxonomically classified. Representatives of the Candida genus were identified in all samples, most notably *C. quercitrusa*, while sequence reads assigned to the Aspergillus genus were only identified in a subset of samples. CloVR-ITS is made available as a pre-installed, automated, and portable software pipeline for cloud-friendly execution as part of the CloVR virtual machine package (http://clovr.org).

**Conclusion:**

The CloVR-ITS pipeline provides fungal microbiota analysis that can be complementary to bacterial 16S rRNA and total metagenome sequence analysis allowing for more comprehensive studies of environmental and host-associated microbial communities.

## Background

The advancement of massively parallel next-generation sequencing technologies has led to the ability to deeply sample complex microbial communities through sequencing of targeted phylogenetic marker genes [1] and whole-genome shotgun DNA [2]. While the 16S ribosomal RNA (rRNA) gene has been a major point of focus for researchers describing bacterial and archaeal communities, significantly less effort has been devoted to markers of fungal organisms and the characterization of fungal components of microbial communities.

The fungal tree of life continues to be explored and refined, and currently, in contrast to 16S rRNA studies, there is little community consensus on conserved sequence markers suitable for broad phylogenetic classifications of eukaryotic organisms or reasonable diversity thresholds to describe eukaryotic taxa at the species or genus-level [3-5]. Recent studies have begun to explore fungal diversity through amplification of the internal transcribed spacer (ITS) region of the eukaryotic rRNA operon [6-16], but there remains a need for community-accepted standards and more rigorous sequence analysis protocols. The internal transcribed spacers ITS1 and ITS2 are the two elements of the eukaryotic rRNA cistron separating the 18S, 5.8S, and 28S rRNA genes, respectively. RNA polymerase 1 synthesizes this cistron as a single long transcript, and internal spacers are subsequently removed from the functional ribosomal RNA elements. Typically, the “ITS region” refers to the contiguous region of ITS1, the 5.8S gene, and ITS2 (see Additional file 1: Figure S1 for a diagram). Because this region can be amplified across a broad range of fungal organisms, using methodologies developed and tested for 16S rRNA amplicon sequence analysis as a reference bears the potential to introduce comparable tools for the characterization of the fungal microbiota [17].

Here we present CloVR-ITS, a standard operating procedure (SOP) for ITS amplicon sequence analysis for fungal diversity studies. This protocol includes preprocessing of multiplexed pyrosequences, *de novo* chimera detection and removal using UCHIME, and high-quality post-filtered sequence alpha and beta-diversity analyses. CloVR-ITS has been designed to effectively process sequence data representing any contiguous segment of the ITS region (i.e. ITS1 or ITS2) and can perform comparative analysis of hundreds of samples. Based on available reference data, we determined robust sequence identity thresholds to approximate the taxonomic distribution and diversity of fungal communities at multiple levels. In order to make our SOP widely available with minimal software installation requirements and capable of running on a cloud-based infrastructure, we have implemented our protocol within the Cloud Virtual Resource (CloVR) framework [18,19]. The CloVR framework guarantees CloVR-ITS users reproducible analysis results, establishes software transparency, and provides cloud-based options for pipeline execution. We illustrate the utility of our pipeline by analyzing fungal ITS data generated from human gastric aspirates of nine patients with variable health status. CloVR-ITS can be run on realistic data in a commercial cloud for a very low price, potentially saving labs the significant investment of additional computing software and hardware.

## Methods

### Taxonomic reference database

To generate a broad ITS reference set for taxonomic assignment of ITS amplicon sequences, we first obtained 213,357 reference ITS2 sequences from the ITS2 database [20,21], together with taxonomic information and corresponding NCBI identification (GI) numbers. The ITS2 database utilizes a comprehensive Hidden Markov Model (HMM) approach for identification of ITS2-specific regions from GenBank sequence entries. Next, full fasta sequence entries from the NCBI-NT database corresponding to the GI numbers were extracted using the *fastacmd* tool [22]. This allows for longer portions of the entire rRNA cistron region (including ITS1, ITS2, 18S, 5.8S, and 28S genes) to be incorporated in the custom database when available. For the final set of annotated sequences, we required sequences not to represent uncultured, environmental, or unclassified taxa. In several cases, the taxonomic lineages of the reference sequences were incomplete, i.e. they were lacking assignments at some of the taxonomic levels (Example: “No class level; Zygophyllales; Krameriaceae; Krameria; Krameria ixine”). In these cases, missing fields in the reference database were filled with, for example, "No_class" or “No_order”. All reference sequences were clustered using a 100% identity threshold to identify identical sequences, which were not removed from the database. The final custom database (named *clovritsdb* version 1.0) consists of 154,050 sequences representing over 8,440 genera and over 60,000 species. The 154,050 sequences belonged to 121,993 clusters that contained between one (107,397 clusters) and 103 identical sequences. In many cases, sequences in a cluster differed in length such that the shorter sequences perfectly matched a subsequence of a longer one. These differences in length can impact the e-values of BLASTN search results used for taxonomic assignment of ITS amplicons (see below). Of the 14,596 clusters with multiple sequences, 4,966 clusters contained sequences with different annotations, typically at the species level; 905 showed differences at the genus level. Use of fasta (sequence) and txt (taxonomic assignments) file formats for clovr-itsdb allows for easy database expansion, as additional ITS amplicon sequence data become available, as well as database curation, e.g. in cases where identical reference sequences have different annotations.

### CloVR-ITS workflow

**Figure 1:**
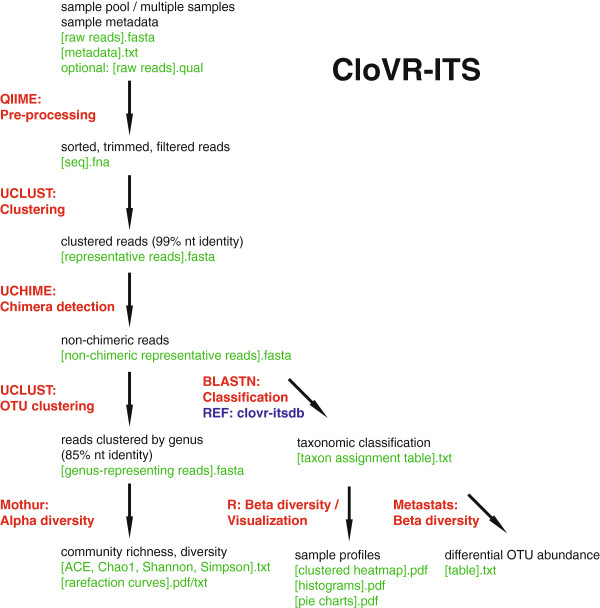
**Flowchart of the CloVR-ITS protocol.** Preprocessing of sequences includes filtering for quality and length, clustering to remove redundant sequence information as well as *de novo* chimera detection. After preprocessing, sequences are clustered into genus-level operational taxonomic units and assigned to a taxonomic lineage using BLASTN with a custom database of reference fungal species (*clovr*-*itsdb v1*.*0*). The pipeline also performs alpha- and beta-diversity analyses including rarefaction curves, computation of ecological estimators, differential abundance detection, and unsupervised clustering of samples according to taxonomic profiles.

The main stages of the CloVR-ITS workflow are summarized below and shown in Figure 1. The standard input file types for the pipeline are the same as commonly used for comparable 16S analysis tools, e.g. QIIME [23]. Input includes sequence fasta files (de-multiplexed, i.e. one file per sample, or multiplexed with sample-specific barcodes), a corresponding tab-delimited metadata mapping file, and (optionally) quality scores. A detailed user-manual, the CloVR-ITS SOP, is available at http://clovr.org/wp-content/uploads/2012/06/CloVR-ITS_20120620.pdf. Several components of the pipeline have been implemented to allow for parallelized execution in a cloud-based environment such as the commercial Amazon Elastic Compute Cloud (http://aws.amazon.com/ec2/) or the free academic Data Intensive Academic Grid (DIAG; http://diagcomputing.org).

#### Preprocessing

CloVR-ITS begins by performing file existence and consistency checks for correct formatting, then de-multiplexing and quality filtering sequences as needed using the QIIME [23] script split_libraries.py with the following non-default parameter choices:

min-seq-length 100 (sets the minimum sequence length to 100 bp)

max-seq-length 2000 (sets the maximum sequence length to 2000 bp)

barcode-type variable_length (disables barcode corrections and allows for unique barcodes with varying lengths)

max-homopolymer 8 (sets the maximum homopolymer length allowed within the sequence to 8 bp)

max-ambig 0 (sets the maximum number of ambiguous bases allowed within the sequence to 0).

As in all CloVR pipelines, parameters can be adjusted by experienced users in the pipeline configuration file as documented on the CloVR project website (http://clovr.org).

#### Chimera detection and removal

To assist in chimera detection and downstream taxonomic analysis, sequences passing quality filtering are clustered into high-similarity groups using a 99% sequence identity threshold with UCLUST [24] (allowing for reverse complement comparisons). The longest element in each cluster is selected as cluster-representing sequence. Formatted representative sequences are then submitted to UCHIME [25] (using *de novo* mode with default parameters). All identified chimeric representatives and their associated sequences are removed from consideration before the next step in the pipeline.

#### Genus-level diversity analysis

Sequences passing the chimera detection step are re-clustered into genus-level operational taxonomic units using UCLUST with an 85% identity threshold, based on our evaluation of available reference data (see below). Using components from the Mothur package [26], alpha-diversity analysis is performed for each sample including computation of rarefaction curves and ecological statistics such as ACE and Chao1 estimators, Shannon entropy, and the Simpson index.

#### Taxonomic and statistical analysis

All non-chimeric representative sequences from the 99% identity clusters are searched against the curated database of ITS reference sequences from known species (*clovritsdb v1*.*0*) using BLASTN [22] with an e-value threshold of 1e-5. Each sequence is assigned to the taxonomic lineage of the best BLAST alignment covering at least 90% of the query sequence length and matching with a minimum identity of 90%, 85%, 75%, 70%, and 60% identity for species, genus, family, order, and class-level assignments, respectively (see justification below). BLAST result tables are provided in the CloVR-ITS pipeline output and contain up to 10 matching reference sequences per query sequence. Representatives without alignments of sufficient coverage or identity are denoted as “Unclassified.” Assignments to representatives are then propagated across the corresponding clusters, so that every clustered sequence is consistently annotated. Classification results are processed to detect differentially abundant taxa between groups of interest at multiple taxonomic levels (class, order, family, genus, species) using the Metastats program [27] with default parameters. Additional visualizations of taxonomic annotations are computed using R-based scripts and include stacked histograms, pie charts, and heatmap clusterings.

### Gastric fluid sample data

ITS1 regions were amplified as part of a larger project to study the microbiota composition of human gastric acid samples (von Rosenvinge *et al*. *in preparation*). The data sets supporting the results of this article are available in the NCBI Short Read Archive repository [SRA: SRA056502; http://www.ncbi.nlm.nih.gov/sra/. Briefly, adult patients with clinically indicated upper endoscopy were enrolled in the study (University of Maryland School of Medicine IRB Protocol #HP-00045881) and gastric fluid aspirated and collected in a sterile container during the procedure. All subjects provided their informed consent. Demographics, clinical features, and endoscopic indications and findings were recorded (Additional file 2: Table S1). Samples were immediately put on ice and within several hours aliquoted (0.5 mL), combined with RNAlater (0.5 mL; Qiagen) and frozen at −80°C for subsequent processing. Samples were centrifuged at 5,000 × g for 8 min and the supernatant discarded. Cell pellets were re-suspended in 0.6 mL of 1X Phosphate Buffered Saline (PBS) and processed as described previously [28]. Cell lysis was initiated with 5 μL of lysozyme (10 mg/mL; Amresco), 13 μL of mutanolysin (11.7 U/μL; Sigma-Aldrich), and 3 μL of lysostaphin (4.5 U/μL; Sigma-Aldrich) and samples incubated at 37°C for 30 min. Samples underwent a second enzyme incubation at 56°C for 45 min, after the addition of 10 μL Proteinase K (20 mg/mL: Research Products International), 50 μL 10% SDS, and 2 μL RNase (10 mg/mL). After the enzyme treatments cells were disrupted by bead beating in tubes with Lysing Matrix B (0.1 mm silica spheres, MP Biomedicals), at 6 m/s for 40 s at room temperature in a FastPrep-24 (MP Biomedicals). The resulting crude lysate was processed using the ZR Fecal DNA mini-prep kit (Zymo) according to the manufacturer's recommendation. The samples were eluted with 100 μL of ultra pure water into separate tubes. The DNA concentrations in the samples were measured using the Quant-iT PicoGreen dsDNA assay kit from Molecular Probes (Invitrogen).

ITS1 regions were amplified with a single standard primer *ITS1F* (*CCTATCCCCTGTGTGCCTTGGCAGTCTCAGCTTGGTCATTTAGAGGAAGTAA*) and barcoded reverse primers *ITS2* (*CCATCTCATCCCTGCGTGTCTCCGACNNNNNNNNGCTGCGTTCTTCATCGATGC*), where the underlined sequence in *ITS1F* and *ITS2* represents the Roche/454 XLR adaptors B and A, respectively, and the *N*'*s* denote variable barcode sequences of seven to eight nucleotides length. These primers were selected for their sensitivity in detection of homologous sequences present in a broad range of fungal species, including those from *Candida* and *Aspergillus* clades [14,16,29,30]. ITS DNA amplification was performed using AccuPrime Taq DNA polymerase High Fidelity (Invitrogen) and 50 ng of template DNA in a total reaction volume of 25 μL, following the AccuPrime product protocol. Reactions were run in a PTC-100 thermal controller (MJ Research) using the following cycling parameters: 2 min of denaturation at 94°C, followed by 35 cycles of 30 s at 94°C (denaturing), 30 s at 50°C (annealing), and 1 min at 68°C (elongation), with a final extension at 68°C for 5 min. Negative controls were included for each PCR amplification and barcoded primer pair, including amplification without template DNA. The presence of amplicons was confirmed by gel electrophoresis on a 2% agarose gel and staining with ethidium bromide. PCR products were quantified using the Quant-iT PicoGreen dsDNA assay. Equimolar amounts (50 ng) of the PCR amplicons were mixed in a single tube. Amplification primers and reaction buffer were removed from each sample using the AMPure Kit (Agencourt). The purified amplicon mixtures were sequenced by 454 FLX XLR pyrosequencing using 454 Life Sciences primer A by the Genomics Resource Center at the Institute for Genome Sciences, University of Maryland School of Medicine, using protocols recommended by the manufacturer as amended by the Center. ITS sequences were screened for human DNA by searching against the NCBI NT database using BLASTN (with word size 26). Six sequence reads from a single patient sample matched human non-ITS DNA with >97% identity along >90% of their length and were removed from the analysis.

### Local and cloud-scale pipeline runs

The local desktop used for CloVR-ITS pipeline tests was a 64-bit quad core (Intel Xeon E5520 2.27 GHz CPU) with 4 GB of RAM. The local version of CloVR (CloVR release candidate *clovr1*.*0RC4*) was run using VMware Player version 2.0.5 build-109488 (http://vmware.com) configured to a single CPU and 2048 MB of main memory. Amazon EC2 allows for a variety of instance types with variable CPU speeds, available RAM and disk storage (http://aws.amazon.com/ec2/#instance). Earlier work has demonstrated that c1.xlarge instances are cost-effective for many bioinformatics applications such as BLAST and HMMER [19,31]. The c1.xlarge instance type provides 8 virtual CPU cores, 7 GB RAM per instance, and 1,690 GB of local temporary disk storage. In this study, each pipeline was run on a separate cluster within the cloud and all instances (master or otherwise) utilized the c1.xlarge instance type. Direct costs of running CloVR-ITS on Amazon EC2 were computed using cluster performance charts, visualized with the Ganglia tool (http://ganglia.sourceforge.net), which describe cluster size and utilization over time. CloVR-ITS pipeline runtimes were obtained using the Ergatis workflow system [32].

To test the scalability of CloVR-ITS using larger datasets, we simulated a 1-million read dataset by randomly sampling sequences (with replacement) from the human gastric fluid dataset, and randomly creating sequencing errors using a simple uniform error rate of 0.8%. This allowed us to create a larger realistic dataset with similar diversity to the original human gastric dataset. For each of 10 samples, 100,000 sequences were simulated.

## Results and discussion

### CloVR implementation

The CloVR-ITS SOP for ITS amplicon sequence analysis for fungal diversity studies was implemented within the Cloud Virtual Resource (CloVR) framework [18,19]. CloVR (http://clovr.org) is an open source virtual appliance that provides automated bioinformatics workflows for microbial genomics applications [18]. CloVR integrates genomic tools in a robust, user-friendly, and fully automated software package with optional support for cloud computing platforms. This tool is distributed as a portable virtual machine that is launched on a desktop or laptop and optionally manages additional resources available on the cloud to perform large-scale sequence analysis. Implementation of the ITS analysis pipeline into CloVR provides the advantage of portability across multiple platforms, access to pre-installed and pre-configured software tools bundled into an automated analysis pipeline, and seamless support of online academic (http://diagcomputing.org) or commercial (http://aws.amazon.com) cloud computing services. Several steps of the CloVR-ITS pipeline are parallelizable (chimera detection, BLAST), allowing for multi-CPU support on a cloud-based infrastructure. In order to take full advantage of the cloud multi-CPU capabilities, the CloVR framework supports auto-scaling for BLAST processes.

### Parameter selection

In an effort to select practical parameter values for our methodology, including sequence similarity thresholds for taxonomic classification, we performed a preliminary diversity study of ITS2 regions from known fungal species, that is, the ITS portion surrounded by the 5.8S and 28S rRNA genes [33]. Our source for reference sequences was the ITS2 database, which utilizes a robust Hidden Markov Model (HMM) based method for identification of ITS2-specific regions [20,21]. We extracted 213,357 taxonomically classified sequences corresponding to 783 genera and 1,898 distinct eukaryotic species.

**Figure 2:**
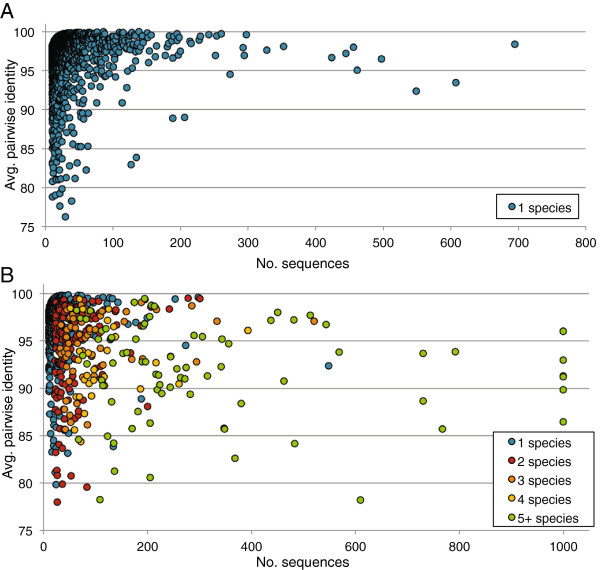
**Pairwise identity of reference ITS sequences within different taxonomic groups.** The plot displays average pairwise identities computed from MUSCLE alignments for groupings of ITS2 reference sequences at the (**A**) species and (**B**) genus level. The y-axis represents the average pairwise identity found between two sequences in the same species (**A**) or genus (**B**), the x-axis represents the total number of sequences from each species (**A**) or genus (**B**) that were aligned, and the color displays the total number of reference species found in each genus (**B**).

From this reference set, we created datasets for each specific taxonomic group ranging from 10 to 1,000 sequences. In cases where more than 1,000 sequences were available for a particular taxon, we randomly selected 1,000 sequences from the full set. Within each taxon grouping, we then performed multiple sequence alignments using MUSCLE with default parameters [34] and computed pairwise identities using custom Perl scripts between all sequences within each MUSCLE alignment to determine the average pairwise identity between any two sequences in the same taxon. Only those columns of the multiple sequence alignment containing <5% gaps were used for the pairwise identity calculations. Figure 2 displays the results of our analysis at the species, genus, and order levels. It is important to note that the amplified ITS fragment contains intergenic regions, which are subject to different selective pressures than rRNA genes and, thus, are expected to evolve at different speeds. Therefore, similarity thresholds typically applied to rRNA genes, e.g. 97% identity for 16S rRNA genes between members of the same species, are not suitable for ITS amplicon sequence analysis.

Overall, we found that in 95% of species, the average pairwise identity between two ITS2 sequences from the same species was greater than 90% (Figure 2A). For more than 95% of all genera, the average pairwise identity within each genus was greater than 85% (Figure 2B). To ensure that an 85% sequence identity threshold provided reasonable separation between most genera, we also computed pairwise identities between randomly selected sequences from different genera using BLASTN. Ultimately the vast majority of genera-specific representatives could not be practically aligned using BLASTN beyond 30 base pairs, indicating substantial sequence heterogeneity, including indels, among ITS2 sequences from different genera. This sequence variation argues against use of tree-based phylogenetic distance predictions that have been employed in 16S rRNA analysis.

However, we did detect reasonable homogeneity among 211 (0.07%) pairwise alignments. Those ITS2 sequences from different genera that aligned over at least 90% of one of the sequences’ lengths, showed a median alignment identity of 91%, which suggests a potential bias toward inadvertent collapse of some genera into clusters. We examined the 201 unique genera that met this alignment criterion, and found that 75% had a significant alignment with only one or two other genera. However in 25 genera (12.4%), we found significant alignments with five to seven other genera. As an example, the representative sequence for the genus Ascochyta had alignments of 98%, 97%, 97%, 96%, 96%, and 96% identity over 90% of the query with representatives for the genera Peyronellaea, Epicoccum, Dothiorella, Stagonosporopsis, Phoma, and Boeremia, respectively. Five of these genera are members of the *Didymellaceae* family, and all are members of the Dothideomycetes class. Examining the overall similarity within each of these genera individually, we observed a range from 82.6% to 99.8%.

These findings clearly demonstrate that based on currently available ITS reference data, a universal set of sequence identity thresholds for taxonomic assignment of ITS amplicons cannot be determined. This could be due either to misclassified entries within the fungal *clovr*-*itsdb* reference database, e.g. incorrect assignment of related species to different genera, or indicate that the fungal ITS region does not provide sufficient resolution to differentiate between several closely related taxonomic groups.

Based on these evaluations, we implemented our ITS protocol to provide reasonable taxonomic resolution and to utilize an 85% identity threshold for clustering sequences into genus-level operational taxonomic units, and to assign sequences to a taxonomic lineage based on similar sequence identity and coverage criteria (see Methods). Our selections are consistent with other validation studies examining species and genus-level diversity using fungal ITS sequences [3,35-38].

### CloVR-ITS performance and cost

To examine the costs and resources needed to run the CloVR-ITS pipeline on a local desktop computer (2048 MB of RAM) and on the commercial Amazon EC2 cloud (c1.xlarge instance type; see Methods for details), we ran an ITS1 amplicon dataset representing fungal communities within a larger human gastric microbiome study (von Rosenvinge *et al*. *in preparation*). Samples from patients averaged 14,965 reads per sample with mean read length of 267 bp and a total number of raw sequence reads of 134,682. CloVR-ITS pipelines completed in less than 2 h on the local desktop and required only 41 min (including data transfer time) to run on Amazon EC2, at a cost of $1.32 in on-demand rental time.

In order to simulate significantly larger CloVR-ITS runs, we also created a dataset of 1 million sequences simulated from our original gastric dataset to run locally and on Amazon EC2 (see Methods for details). Including data transfer time, CloVR-ITS required 3 hours 23 minutes to complete locally, and less than 2 hours when the pipeline was executed on Amazon EC2. Importantly, CloVR-ITS auto-scaled from a single instance on Amazon EC2 to a two-node cluster with a total of 16 CPUs during the chimera detection and BLAST steps of the pipeline. The overall cost from start to finish for this run was $1.98, indicating that even larger datasets can be analyzed by CloVR-ITS at negligible cost in a commercial cloud environment.

### Analysis of gastric fluid ITS dataset

**Figure 3:**
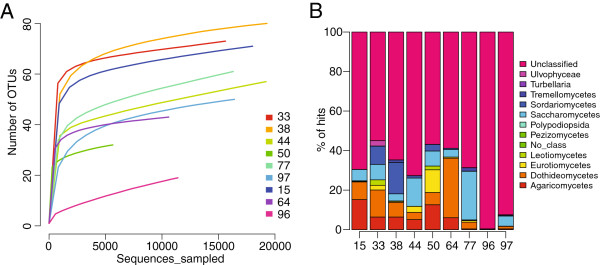
**CloVR-ITS rarefaction and histogram visualizations for gastric fluid data.** Example outputs of the CloVR-ITS pipeline for the human gastric fluid data: (**A**) rarefaction curves of genus-level OTUs (85% identity threshold for clustering) and (**B**) stacked histograms displaying relative abundances at the taxonomic class level (60% identity threshold over 90% of the sequence).

**Figure 4:**
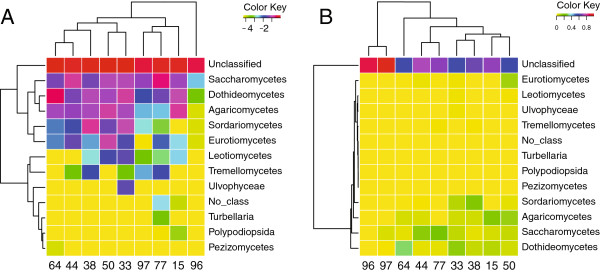
**CloVR-ITS heatmap clustering visualizations for gastric fluid data.** CloVR-ITS outputs hierarchical clusterings of samples and annotations at several taxonomic levels. These clusterings are computed utilizing the furthest-neighbor algorithm with a Euclidean-distance metric on either log-transformed proportions or simple proportions of taxon groups, shown at the class level in (**A**) and (**B**), respectively. While proportions alone help the user assess the overall distribution of taxonomic assignments for each sample, log-transformed proportions can be useful for visualizing similarities or differences between samples that affect members of the community that may make up less than 1% of the total sequences in a sample. Clusterings did not demonstrate significant associations among gastric fluid samples from subjects with similar treatments or conditions (see Additional file 2: Table S1 for sample metadata).

To illustrate the utility of our pipeline, we describe the CloVR-ITS analysis results of the previously mentioned human gastric fluid study (von Rosenvinge *et al*. *in preparation*). For this study, stomach fluid was collected from a diverse patient population and used for DNA isolation and ITS1-specific PCR amplification (see Additional file 1: Figure S1 for a diagram of primer locations). A summary of the patient metadata is given in Additional file 2: Table S1. All results described in this section are based on outputs generated directly from the CloVR-ITS pipeline including numerous visualizations (see Figures 3 and 4).

Of 134,682 total raw DNA sequences used as input to the pipeline, 133,763 (99.3%) passed initial quality checking. Using UCHIME for chimera assessment (see Methods), a total of 1,235 reads were identified as chimeric and removed by the pipeline. Table S2 describes the overall summary statistics of quality-filtering and chimera detection for each sample (see Additional file 3: Table S2). After trimming of primer and barcode sequences, amplicon sizes ranged from 71 to 569 bp (median trimmed length 253 bp), with all samples showing a similar distribution of size ranges, consistent with the visual control of the ITS amplification results using gel electrophoresis prior to sequencing. Passing sequences (average 14,725 per sample) were clustered into 195 genus-level OTUs using the 85% identity threshold; six of these 195 OTUs were found in all nine patient samples.

Rarefaction analysis revealed a substantial range of diversity among patient samples (Figure 3A) with a sample from a HIV/AIDS patient (#96) treated prophylactically with trimethoprim/sulfamethoxazole (co-trimoxazole) against *Pneumocystis* pneumonia (PCP) exhibiting the least diverse fungal microbiota. Co-trimoxazole is an effective broad-spectrum antibiotic, which also exhibits anti-fungal activities [39]. Reduced complexity of the fungal microbiota in this patient sample was also indicated by ecological estimators computed by the CloVR-ITS pipeline (e.g. Chao1 and ACE richness estimators, and the Shannon diversity index; see Table 1).

**Table 1:** Ecological estimators of alpha-diversity for human gastric fungal communities, including 95% confidence intervals (shown in parentheses)

**Subject**	**Genus-level OTUs**	**Chao**	**Ace**	**Shannon**
15	71	82.3 (73.7, 118.7)	124.7 (98.3, 176.6)	3.00 (2.98, 3.02)
33	72	100 (79.3, 179.0)	91.7 (80.0, 120.2)	3.5 (3.44, 3.47)
38	81	90.2 (83.3, 117.7)	97.9 (88.3, 120.0)	2.99 (2.98, 3.01)
44	57	117 (73.7, 273.2)	247.1 (179.1, 352.9)	2.66 (2.65, 2.68)
50	32	34 (32.2, 48.0)	39.3 (34.1, 57.1)	2.76 (2.74, 2.79)
64	42	52 (43.9, 95.6)	51.5 (44.9, 73.0)	2.28 (2.25, 2.31)
77	61	121 (77.7, 277.2)	134.8 (103.5 189.1)	2.17 (2.15, 2.19)
96	19	37.3 (23.5, 94.1)	40.1 (24.3, 102.4)	0.14 (0.13, 0.16)
97	51	55.5 (52.0, 71.7)	58.7 (53.3, 76.9)	0.70 (0.67, 0.72)

CloVR-ITS calculates estimators for each sample independently without controlling for sampling depth across all samples.

Using the BLAST-based CloVR-ITS taxonomic classification of representative ITS amplicons clustered at 99% identity (see Methods for details), we found all samples to be dominated by sequences that could not be confidently assigned to a taxonomic lineage. On average 71% of these representative sequences within a sample could not be classified (Figure 3B). Of the taxonomically-assigned sequences, the largest fractions belonged to Dothideomycetes and Saccharomycetes (both average 8%), Agaricomycetes (average 6%) and Sordariomycetes (average 3%) (see Additional file 4: Table S3 for full results).

To further test how well the variation in the gastric ITS amplicon sequence data was represented by the clovr-itsdb v1.0 reference database, sequence coverage and identity values of top CloVR-ITS BLAST matches were plotted (see Additional file 5: Figure S2). The majority of all reads (84%) produced significant BLAST hits (e-value threshold: 1e-5), in most cases with >80% query coverage (67% of all top BLAST hits). To test whether non-matching reads or reads producing low-identity hits also represented ITS regions, all reads representative of 99% clusters were also searched by BLAST against NCBI NT (e-value threshold: 1e-5). Manual inspection of these BLAST hits suggested that the vast majority matched fungal 18S rRNA/ITS spacer regions when searched against NCBI NT, supporting the notion that the ITS1F/ITS2 amplification products were generally specific to the ITS region.

As part of its graphical output, CloVR-ITS produces heatmaps with hierarchical clusterings of taxonomic abundances using either standard relative abundances or log-normalized proportions. Log-normalized proportions provide the advantage of (i) allowing for more equivalent weighting of high- and low-abundance taxa for clustering purposes, and (ii) making differences between low-abundance taxa more easily visible (compare Figure 4A and B). Clustering samples based on taxonomic abundances, we observed different groupings depending on whether or not we used a log-normalized transformation (Figure 4). Clustering did not reveal obvious correlations between fungal microbiota compositions and specific patient groups (see Additional file 2: Table S1 for clinical features of subjects). However, statistical validation of the results is difficult due to low sample numbers and highly variable patient backgrounds.

At lower taxonomic levels we found substantial disparities in diversity among the samples. A total of 12 classes, 30 orders, 47 families, 82 genera, and 143 species were assigned by our BLAST-based procedure (see Additional file 4: Table S3). At the species level, sequences from each subject could be assigned to Candida species, particularly to the species *C*. *quercitrusa* (Table 2); the sample from the HIV/AIDS patient treated with co-trimoxazole was the only patient sample without significant *C*. *quercitrusa* content (1 sequence). This sample, however, contained 32 *C*. *dubliniensis* sequences, a species which was only found in one other patient sample (#77) at low counts (1 sequence). Although we cannot provide robust statistical support for the association of *C*. *dubliniesis* with HIV/AIDS gastric fluid samples, it is intriguing that *C*. *dubliniensis* is a less virulent and less metabolically versatile close relative of *C*. *albicans*[40] which has been associated with bloodstream and other infections particularly in immunocompromised individuals, including HIV/AIDS patients [41]. We also found differences in Aspergillus abundance (Table 3), with several samples being devoid of Aspergillus assignments altogether (such as the low-diversity sample #96), while in others between 0.3 and 1.2% were assigned to different Aspergillus species including *A*. *versicolor*.

**Table 2:** Overview of sequence reads from all samples assigned to known Candida species

	**Patient samples**								
	**#15**	**#33**	**#38**	**#44**	**#50**	**#64**	**#77**	**#96**	**#97**
**Candida (genus)**	995	1193	700	2632	159	401	4008	33	841
*C*. *albicans*	8	0	42	0	0	202	2	0	509
*C*. *dubliniensis*	0	0	0	0	0	0	1	32	0
*C*. *ergatensis*	1	0	0	0	0	0	0	0	0
*C*. *metapsilosis*	0	32	0	0	0	0	0	0	0
*C*. *parapsilosis*	0	0	2	57	0	1	2	0	1
*C*. *quercitrusa*	986	1107	656	2573	159	198	3999	1	331
*C. sp. 3TMS-2011*	0	54	0	0	0	0	0	0	0
*C. sp. JJP-2009a*	0	0	0	0	0	0	1	0	0
*C. tropicalis*	0	0	0	2	0	0	3	0	0

**Table 3:** Overview of sequence reads from all samples assigned to known Aspergillus species

	**Patient samples**								
	**#15**	**#33**	**#38**	**#44**	**#50**	**#64**	**#77**	**#96**	**#97**
Aspergillus (genus)	46	87	0	52	70	0	8	0	1
*A. penicillioides*	1	0	0	0	0	0	0	0	0
*A. ruber*	0	0	0	0	0	0	1	0	0
*A. sp. 6 SMR-2010*	0	0	0	0	0	0	0	0	1
*A. sp. F55*	0	86	0	19	0	0	0	0	0
*A. sp. F9*	0	1	0	0	0	0	0	0	0
*A. versicolor*	45	0	0	33	70	0	7	0	0

Overall, based on the species-level classification, CloVR-ITS analysis of the gastric fluid samples suggests that fungal species in the stomach most likely originated from environmental sources, such as mold and degrading plant material, e.g. *Cladosporium* spp., *Aureobasidium pullulans*, *Schizophyllum commune*, which were found in nine, eight and six of the nine samples, respectively. It is possible that at least some of the fungal species identified in the stomach were originally collected from the environment through the respiratory tract or ingested via vegetable food components.

## Conclusions

CloVR-ITS is the first automated comparative analysis protocol for ITS amplicon pyrosequencing-based fungal microbiota analysis. Implemented in the CloVR virtual machine (http://clovr.org), it can be run time- and cost-efficiently in cloud-based infrastructures. Similarity thresholds for the assignment of ITS amplicons to known taxonomic lineages have been determined and included into CloVR-ITS based on the evaluation of available fungal sequence data. As an example for the utility of CloVR-ITS, we have presented the analysis of human gastric fluid samples, which demonstrates that fungal species typically associated with environmental or plant and human host settings most likely collected through food, the nasal and oral cavities and the lungs can be identified in the stomach. Large fractions of unclassified gastric fluid ITS amplicon sequence data suggest that the fungal microbiota of the stomach and probably also of other human-associated environments is still mostly underrepresented in available ITS sequence collections. The CloVR-ITS pipeline provides complementary analysis for bacterial 16S rRNA and total metagenome sequence analysis protocols such as CloVR-16S and CloVR-Metagenomics [19], QIIME [23], MG-RAST [42], Galaxy [43] and others, allowing for broader studies of environmental and host-associated microbial communities. We expect to refine and expand CloVR-ITS as the research community adopts more standards regarding ITS sequence analysis of fungal communities.

## Availability of supporting data

The data sets supporting the results of this article are available in the NCBI Short Read Archive repository [SRA: SRA056502; http://ncbi.nlm.nih.gov/sra/].

## Competing interests

The authors declare that they have no competing interests.

## Authors’ contributions

JRW and WFF conceived the study design. CM performed the experimental work and provided technical support. JRW carried out the implementation and testing. JRW and WFF performed the analysis. SVA provided technical support. JRW and WFF wrote the manuscript with help from CM, OW, and SVA. All authors read and approved the final manuscript.

## Supplementary Material

Additional file 1 **Figure S1.** Overview of the ITS region and primers used in this study. Within the eukaryotic rRNA cistron, the genes coding for 18S, 5.8S, and 28S rRNA are separated by two transcribed spaces (ITS1 and ITS2). RNA polymerase 1 synthesizes this cistron as a single long transcript, and internal spacers are subsequently removed from the functional ribosomal RNA elements. In this study, we refer to the “ITS region” as the contiguous region of ITS1, the 5.8S gene, and ITS2. The human gastric fluid dataset analyzed in this paper were amplified with the ITS1/ITS2F primer pair encompassing the ITS1 region. Click here for file

Additional file 2 **Table S1.** Demographics and clinical features of the enrolled patients. This table gives an overview of the patient demographic and clinical feature metadata associated with the human gastric fluid samples analyzed in this paper. Click here for file

Additional file 3 **Table S2.** Pipeline sequence analysis statistics for human gastric ITS dataset. This table provides an overview of the number of sequences from each human gastric fluid sample that passed CloVR-ITS quality filtering and chimera detection stages. Click here for file

Additional file 4 **Table S3.** ITS taxonomic assignment tables. Summary tables of ITS taxonomic classifications for human gastric fluid samples. Click here for file

Additional file 5 **Figure S2.** Query coverage versus alignment identity for top-scoring BLASTN hits against clovr-itsdb v.1.0 for high-identity cluster representatives from the human gastric fluid dataset. High-identity (99% threshold) cluster representatives were searched against clovr-itsdb v.1.0 to determine the potential for taxonomic assignment. Here we plot the alignment identity and query coverage of the top-scoring hit for each representative. Click here for file
