# Supplementary figures and images for: CloVR-ITS: Automated internal transcribed spacer amplicon sequence analysis pipeline for the characterization of fungal microbiota
Source: Microbiome. 2013 Feb 4;1:6. doi: 10.1186/2049-2618-1-6 (PMC3869194; doi:10.1186/2049-2618-1-6)

**Figure S1. Overview of the ITS region and primers used in this study.**

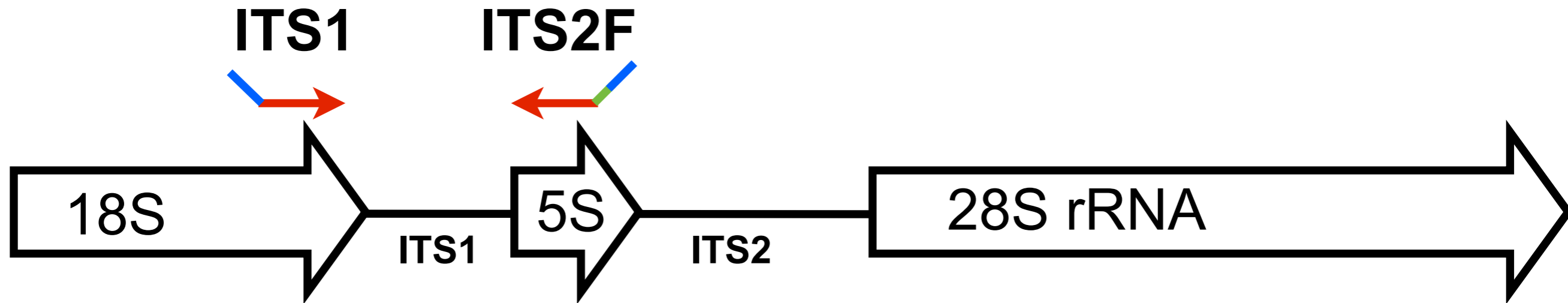

Supplement: Additional file 1 — Figure S1. Overview of the ITS region and primers used in this study. Within the eukaryotic rRNA cistron, the genes coding for 18S, 5.8S, and 28S rRNA are separated by two transcribed spaces (ITS1 and ITS2). RNA polymerase 1 synthesizes this cistron as a single long transcript, and internal spacers are subsequently removed from the functional ribosomal RNA elements. In this study, we refer to the “ITS region” as the contiguous region of ITS1, the 5.8S gene, and ITS2. The human gastric fluid dataset analyzed in this paper were amplified with the ITS1/ITS2F primer pair encompassing the ITS1 region. [file 2049-2618-1-6-S1.pdf]
