# Supplementary material for: CloVR-ITS: Automated internal transcribed spacer amplicon sequence analysis pipeline for the characterization of fungal microbiota
Source: Microbiome. 2013 Feb 4;1:6. doi: 10.1186/2049-2618-1-6 (PMC3869194; doi:10.1186/2049-2618-1-6)

**Figure S2: Query coverage versus alignment identity for top-scoring BLASTN hits against clovr-itsdb v.1.0 for high-identity cluster representatives from the human gastric fluid dataset.**

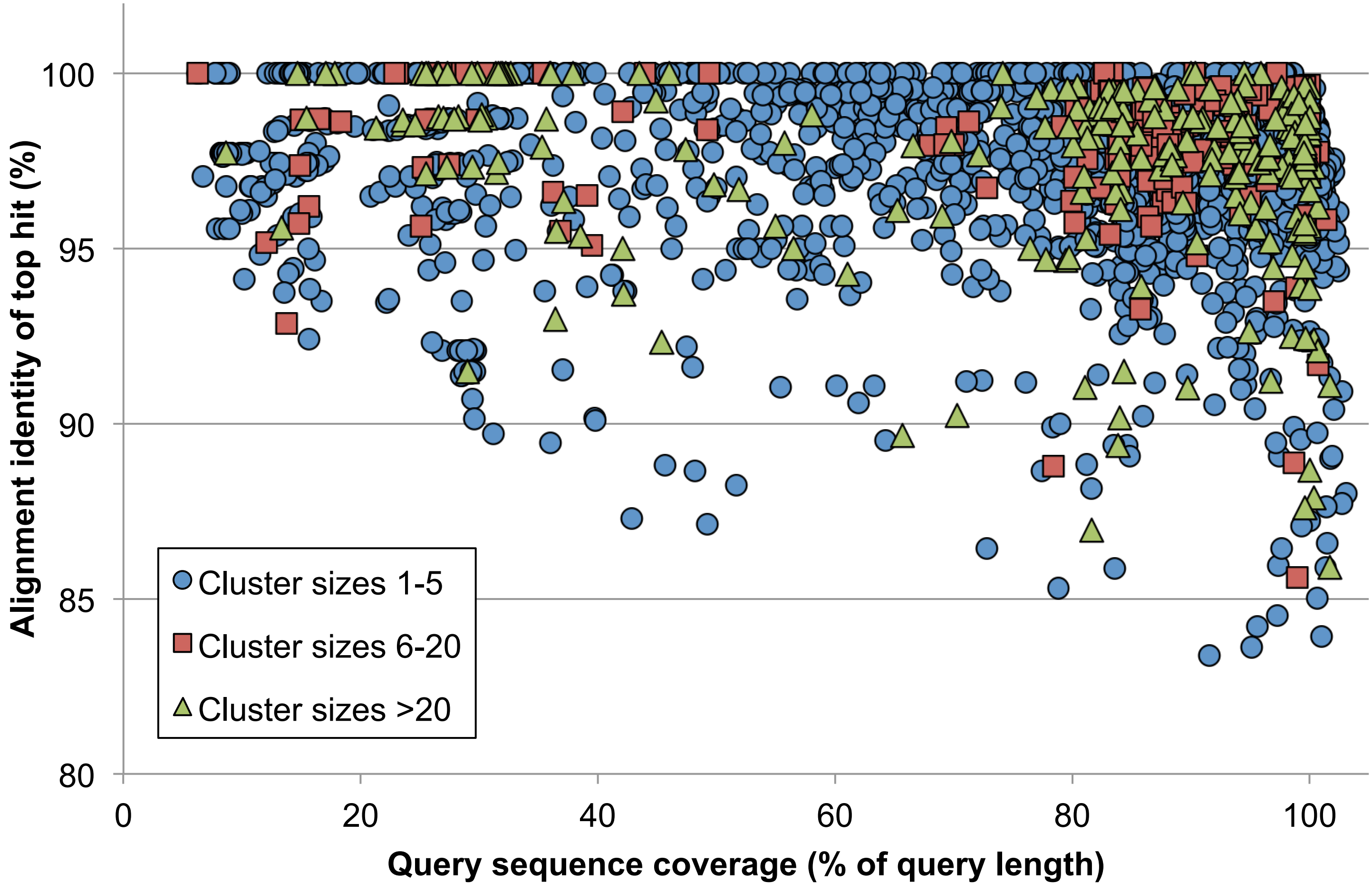

Supplement: Additional file 5 — Figure S2. Query coverage versus alignment identity for top-scoring BLASTN hits against clovr-itsdb v.1.0 for high-identity cluster representatives from the human gastric fluid dataset. High-identity (99% threshold) cluster representatives were searched against clovr-itsdb v.1.0 to determine the potential for taxonomic assignment. Here we plot the alignment identity and query coverage of the top-scoring hit for each representative. [file 2049-2618-1-6-S5.pdf]
